# Biosensors for the Determination of SARS-CoV-2 Virus and Diagnosis of COVID-19 Infection

**DOI:** 10.3390/ijms23020666

**Published:** 2022-01-08

**Authors:** Maryia Drobysh, Almira Ramanaviciene, Roman Viter, Chien-Fu Chen, Urte Samukaite-Bubniene, Vilma Ratautaite, Arunas Ramanavicius

**Affiliations:** 1 State Research Institute Center for Physical and Technological Sciences, Sauletekio Ave. 3, LT-10257 Vilnius, Lithuania; maryia.drobysh@ftmc.lt (M.D.); urte.samukaite-bubniene@chf.vu.lt (U.S.-B.); vilma.ratautaite@ftmc.lt (V.R.); 2 NanoTechnas—Center of Nanotechnology and Materials Science, Faculty of Chemistry and Geosciences, Vilnius University, Naugarduko Str. 24, LT-03225 Vilnius, Lithuania; almira.ramanaviciene@chf.vu.lt; 3 Center for Collective Use of Scientific Equipment, Sumy State University, Sanatornaya Str. 31, 40018 Sumy, Ukraine; 4 Institute of Atomic Physics and Spectroscopy, University of Latvia, Jelgavas Street 3, LV-1004 Riga, Latvia; 5 Institute of Applied Mechanics, National Taiwan University 1, Sec. 4, Roosevelt Rd., Da’an Dist., Taipei 106, Taiwan; stevechen@iam.ntu.edu.tw

**Keywords:** COVID-19, SARS-CoV-2 virus, biosensors, electrochemical immunosensors, bioelectrochemistry, RNA analysis, antigen-antibody interaction, immune complex, immobilisation of biomolecules, molecularly imprinted polymers (MIPs)

## Abstract

Monitoring and tracking infection is required in order to reduce the spread of the coronavirus disease 2019 (COVID-19), induced by severe acute respiratory syndrome coronavirus 2 (SARS-CoV-2). To achieve this goal, the development and deployment of quick, accurate, and sensitive diagnostic methods are necessary. The determination of the SARS-CoV-2 virus is performed by biosensing devices, which vary according to detection methods and the biomarkers which are inducing/providing an analytical signal. RNA hybridisation, antigen-antibody affinity interaction, and a variety of other biological reactions are commonly used to generate analytical signals that can be precisely detected using electrochemical, electrochemiluminescence, optical, and other methodologies and transducers. Electrochemical biosensors, in particular, correspond to the current trend of bioanalytical process acceleration and simplification. Immunosensors are based on the determination of antigen-antibody interaction, which on some occasions can be determined in a label-free mode with sufficient sensitivity.

## 1. Introduction

In March 2020, the worldwide coronavirus disease 2019 (COVID-19) pandemic was proclaimed. The major danger posed by the pandemic is the overburdening of healthcare systems. The most effective method to prevent the spread of the severe acute respiratory syndrome coronavirus 2 (SARS-CoV-2) causing the illness, is to reduce the rate of transmission which can be accomplished by fast monitoring carriers of SARS-CoV-2. Therefore, the diagnosis of COVID-19 is the first step toward effective control of this disease. Thus, the design and implementation of fast, accurate, and sensitive procedures for the detection of coronaviral infection are needed. 

SARS-CoV-2 is a coronavirus of a spherical shape and diameter of around 130 nm [1,2,3] with ‘spike-like structures’ all over its surface. A nucleocapsid carrying positive-sense, single-stranded RNA (ssRNA), the virus genetic information carrier, is located within the viral particle (VP). SARS-CoV-2 contains a genome that is typical for most coronaviruses, specifically, severe acute respiratory syndrome coronavirus (SARS-CoV) and middle east respiratory syndrome coronavirus (MERS-CoV) by roughly 80% and 50%, respectively [4]. The genome encodes structural spike (S), envelope (E), nucleocapsid (N), and membrane (M) proteins [4] (Table 1). The S-protein, which is a transmembrane homo-trimer, is crucial for the virus adhesion and infection of a host cell [5,6]. This protein is formed of two subunits, S1 and S2 [4,7,8]. The receptor-binding domain (RBD) located on the S1 subunit attaches to a host receptor, while the S2 subunit provides the viral and host membrane fusion [9,10,11,12]. The viral envelope is formed by the lower component of the E-protein produced in invaded host cells, whereas the larger component participates in the viral assembling and maturing [13,14]. The N-protein is responsible for virion production by binding to a viral RNA [15] and includes an amino-terminal domain (NTD) and a carboxyl-terminal domain [15,16,17]. The M-protein takes part in the structure of the viral envelope [18].

After infection, SARS-CoV-2 attaches to the host cell receptor, angiotensin-converting enzyme 2 (ACE2), by the RBD, with subsequent fusion with the cell membrane and viral genome injection into the cytoplasm [4,19]. Later, the structural proteins are translated and transferred into the endoplasmic-reticulum–Golgi intermediate compartment [20,21]. Afterwards, N-protein forms the nucleocapsid of the viral genome, and the M-protein manages the protein-protein interactions forming the VP. Eventually, virions are transferred to the cellular surface followed by exocytosis [4,15]. In our previous work, the SARS-CoV-2 life cycle was reviewed in more detail [22].

When SARS-CoV-2 enters the body, an immunological response is triggered [23] and a sequential stimulation of various immune cells results in the induction of the release of antigen-specific antibodies, mainly immunoglobulins M and G (IgM and IgG), which are specific indicators of coronavirus infection [24]. IgM peaks 2–5 weeks after infection, but IgG peaks later, after 3–7 weeks, and remains reasonably steady for up to 105 days post-symptom onset [25,26]. The S- and N-proteins serve as antigens for specific binding to antibodies [27]. 

**Table 1:** Location, mass, and function of SARS-CoV-2 structural proteins.

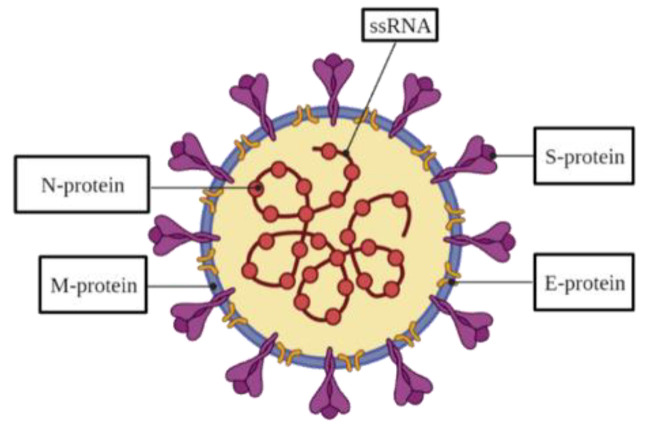	**Protein**	**Mass**	**Function**
	S-protein	∼180 kDa [7]	Accession and infection of a host cell.
	E-protein	∼10 kDa [28]	Viral envelope formation. Assembly and development of the virus.
	N-protein	∼45–60 kDa [15]	Virion shaping.
	M-protein	∼25–30 kDa [29]	Formation of the viral envelope.

## 2. COVID-19 Diagnosis

**Figure 1:**
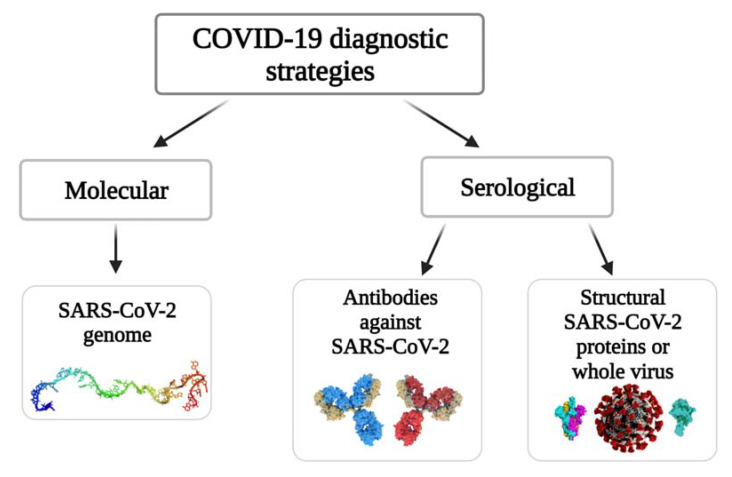
The strategies of COVID-19 diagnosis.

Generally, the COVID-19 diagnostic strategies can be divided into two main groups according to the target compounds, namely, molecular and serological (Figure 1). Molecular tests (so-called ‘molecular assays’) are based on viral RNA determination and allow for spotting the current presence of the SARS-CoV-2 in the host organism. In serological tests, the affinity interaction between antigens (structural proteins of SARS-CoV-2) and specific antibodies is exploited for the determination of infection. In the case of the determination of the specific antibodies, the serological approach allows monitoring the stages of the disease and/or identification/determination of how the organism of the patient has developed immunity against this viral infection. With the detection of SARS-CoV-2 proteins, which are acting as antigens for specific antibodies, it is possible to identify the presence of active viral infection which marks this as an alternative to some molecular assay-based methods.

In addition, biosensors can be used to detect SARS-CoV-2 infection [30]. Several kinds of signal conversion systems are used for the development of biosensors, including electrochemical, optical, and many others. Electrochemical biosensors are mostly used for biomedical purposes because of their low cost, ease of application, and suitability for mass production [30]. Considering the poor electrochemical activity of target biomolecules, there is a necessity to use additional labels (e.g., redox probes) in order to obtain and amplify the analytical signal for some types of biosensors. However, as will be shown below, there are many examples of label-free electrochemical-based sensors, which is one of their main advantages.

### 2.1. Molecular Tests

Up to the present time, the so-called ‘gold standard’ testing method consists of the detection of a specific sequence of SARS-CoV-2 RNA using the reverse transcription-polymerase chain reaction (RT-PCR) [31]. The approach is based on RNA reverse transcription into complementary DNA (cDNA), cDNA amplification, and quantitative RT-PCR detection [32]. Signal registration can be accomplished by monitoring the reaction’s active and continuing status (in real-time) or by performing post-reaction analysis. Quantitative RT-PCR detection time is roughly 1 h and a limit of detection (LOD) is 689.3 copies/mL [33]. The primary drawbacks of this strategy are the high cost of the equipment and the demand for highly skilled analytical staff [34]. 

Reverse transcription loop-mediated isothermal amplification (RT-LAMP) is an alternative extensively utilised molecular technique. LAMP is an amplification approach that relies on the 5′→3′ exonuclease activity of *Taq* DNA polymerase enzyme in targeted multiple primers, each of which recognises different segments of the target DNA [35]. By using a composite LAMP tactic based on reverse transcriptase, the RT-LAMP test allows the carrying out of simultaneous RNA into DNA transcription and the ‘amplification’ of formed DNA [34]. With the LOD of 200 specific RNA sequences in one mL of the aliquot (copies/mL), RT-LAMP takes ~30 min per test [36]. The RT-LAMP techniques offer some advantages: (i) overall cost of the reagent is low, which are determined by skipping several stages that were typically applied in quantitative RT-PCR technique, (ii) time required for the comprehensive performance is reduced to avoid the contamination risk, (iii) the demand for equipment is low, for example, there is no need for precise temperature control based on the isothermal heating process. However, the stability of the LAMP primers-set designed remains extremely challenging in this strategy [34]. 

Recombinase polymerase amplification (RPA) is one more widely used isothermal amplification approach. The reaction mechanism of RPA is employed by three kinds (two complementary) of primers for a DNA rapid extension system [37]. It starts the complex formation of recombinase binding with primers, which detect the homologous sequences in double-stranded DNA. After the primers perfectly match with the complementary sequence, a strand exchange reaction occurs, and single-stranded binding proteins align to the unwound DNA strand, followed by the DNA emerges exponential amplification [38,39]. The RT-RPA assay has high sensitivity and specificity, that efficiency was equal to real-time RT-PCR. On this basis, the innovation method applied for the SARS-CoV-2 rapid detection assay resulted in facilitating better detection times within 20 min and achieving the LOD of 5 copies/μL [40,41]. According to the optimal reaction temperature (from 37 to 42 °C), RPA was suitable to cooperate with clustered regularly interspaced short palindromic repeats (CRISPR) assay in the molecular diagnosis field. The prior study showed that the high-efficiency amplification of RT-RPA followed by Cas12a trans-cleavage processed could achieve the SARS-CoV-2 LOD to 1 copy per test [42].

The CRISPR-based technique is one of the most modern approaches to SARS-CoV-2 detection. The main idea of this method is the utilisation of Cas12/Cas13 enzymes that are related to RNAs and were first discovered as a part of the ‘bacterial immune system’, with subsequent selective binding to a particular area of target DNA or RNA [43]. DNA endonuclease-targeted CRISPR Trans Reporter (‘DETECTR’) and Specific High-Sensitivity Enzymatic Reporter UnLocking (SHERLOCK) testings in one pot COVID (‘STOPCovid’) are the two most shared SARS-CoV-2 detection approaches. The Cas12a enzyme is used in the ‘DETECTR’, which targets the SARS-CoV-2 E- and N-genes, whereas the Cas12b enzyme is used in the ‘STOPCovid’ test, which targets the N-gene. The basic concept is the same for both techniques: the first phase consists of the use of RT-LAMP, then the use of Cas12 enzymes to cleave target compounds and signal registration using a lateral flow or fluorescence assay [43,44]. The ‘DETECTR’ and ‘STOPCovid’ detection time/LODs are ∼30 min/10 copies/µL [45] and ∼40 min/2 copies/µL [46], respectively. Our earlier review article provides a more complete summary of various DNA-enzyme-based techniques, as well as those that use the CRISPR-Cas system [47]. 

#### 2.1.1. Electrochemical Biosensors for SARS-CoV-2 RNA Detection 

**Figure 2:**
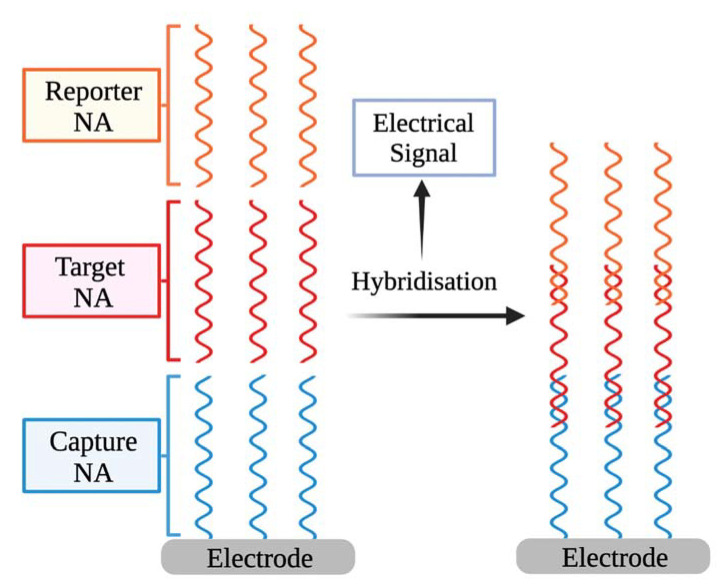
The basic outline of electrochemical biosensors for detecting NA sequences.

As an electrochemical signal source, an electrochemical biosensor uses the hybridisation of single-stranded nucleic acid (NA) with the complementary strands [48]. The biorecognition element of the biosensor consists of the capture of NA engaged particularly with the target NA and an analytical signal converter that converts the recognition event into a measurable electrical signal [49] (Figure 2). The biosensors operating concept is based on the recognition of hybridisation of two complementary NA strands [50,51]. Additional reporter probes that are labelled with signalling substances can be employed. Hybridisation might take place on an electrode surface or in a solution [52].

Detection of NA hybridisation involves an electrochemical reaction in some electrochemical-based biosensors [48], which is hereinafter utilised to quantify the observed NA fragment concentration and hence the SARS-CoV-2 VPs concentration. NA reporter types (labelled/label-free) and the signal production technique (reagent-free/dependent) are used to categorise electrochemical NA biosensors [53].

The critical aspect for electrochemical NA biosensors is to identify selectively a small amount of DNA/RNA copies in samples. The important feature that is employed to resolve this problem is the selection of the most effective signal amplification method. The molecular techniques are divided into three categories. The first one is enzyme-mediated isothermal amplification (NA-based amplification). In the second technique, nanomaterials are used as reporter probes (nanomaterials-based amplification). In another method, enzymes are associated with the NA hybridisation system (enzyme-mediated signal amplification) [53]. For the quantification of amplified signals, several electrochemical approaches are used, in particular cyclic voltammetry (CV) [54,55], square wave voltammetry (SWV) [56], chronoamperometry [57], electrochemical impedance spectroscopy (EIS) [58,59], differential pulse voltammetry (DPV) [60], and pulsed amperometric detection [48,61].

A DPV-based technology for targeting SARS-CoV-2 RNA using graphene oxide functionalised with calixarene was reported [62]. It was confirmed that by means of a compact electrochemical smartphone, the technology can identify the RNA of SARS-CoV-2 without the need for amplification or reverse transcription. A capture, label, and auxiliary probe and target sequence are applied in the design of this biosensor [63]. The capture probe complements the target sequence’s 5′ terminus, whilst the label probe complements the 3′ terminus; two separate label probe locations contain complementary sequences to the auxiliary probe’s 5′ and 3′ regions [63,64]. Every label probe was typically labelled with only one signal molecule, resulting in a weak current signal. As a result, it has been proposed [62] that the relocation of the label probe of signalling compounds to other materials or substances might positively affect sensitivity. The LOD and the sensitivity of the method are 200 copies/mL and 85.5%, respectively. Detection of SARS-CoV-2 RNA was performed in different clinical samples, such as saliva, sputum, etc. [62].

Rolling circle amplification (RCA) is widely utilised for nucleic acid testing [65]. The RCA approach entails annealing DNA or RNA primers to a circular DNA template using polymerases [66]. With few reagents, RCA can synthesise 10^9^-fold concatemers comprising numerous repetitions of complementary sequences to the circular template (amplicons) within 90 min [67]. An electrochemical biosensor may be used to detect the amplicons, which is a key benefit of RCA [68]. For the fast SARS-CoV-2 N- and S-genes detection in patent samples, an electrochemical biosensor based on multiplex RCA was developed. A sandwich hybridisation method based on a three-DNA-component of RCA amplicons with probes functionalised with redox-active dye is used in the experiment followed by the detection using DPV. The method enables determining as low as 1 copy/μL of viral N- or S-genes within less than 2 h from nasopharyngeal swabs [68].

Recently, an electrochemical biosensor based on graphene equipped with an electrical output system suitable for selective SARS-CoV-2 genetic material detection was developed [69]. The biosensor selectivity stems from the incorporation of an appropriate design of thiol-modified antisense single-stranded oligonucleotides (ssDNA) specific for the SARS-CoV-2 N-gene. To improve the analytical performance of the approach, four ssDNA probes were created to simultaneously target two distinct regions in the same viral N-gene [70]. The probes were immobilised on the surface of a paper-based platform and a handmade device including a microcontroller and a signal conditioner circuit was used to collect current-voltage electrochemical data. In addition, thiol-modified ssDNA-capped gold nanoparticles (AuNPs) were applied to further increase the assay sensitivity [69]. It was found that the SARS-CoV-2 can be identified within 5 min incubation in RNA-target containing samples. The sensitivity and LOD of the assay are 231 copies/μL and 6.9 copies/μL, respectively, in nasal swab or saliva samples [69]. 

#### 2.1.2. Electrochemiluminescence-Based Biosensors

**Figure 3:**
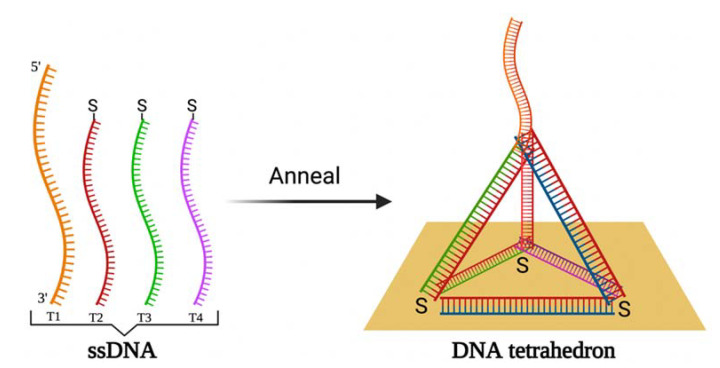
The schematic illustration of DNA tetrahedron formation by annealing of four ssDNA strains followed by the immobilisation DNA tetrahedron on the gold electrode surface.

An electrochemiluminescence (ECL)-based biosensor can be used for the detection of the SARS-CoV-2 RNA-dependent RNA polymerase (RdRp) gene. In ECL, an enzyme-free entropy-driven reaction occurs on the framework represented by a DNA tetrahedron on the gold electrode surface [71]. The DNA tetrahedron is a nanoparticle with a three-dimensional geometric structure with four points and six single strands. It is applied as a carcass on the electrode surface for electrochemical and ECL measurements [72], instead of conventional linear ssDNA or double-stranded DNA due to the simple synthesis and structural rigidity of the DNA tetrahedron [73,74], which improves biosensor stability. The DNA tetrahedron was produced by annealing a DNA strand with 98 nucleotides (T1) and 3 59-nucleotide thiolated DNA strands (T2, T3, T4) and immobilised on a gold electrode surface (Figure 3). Initially, the capture NA probe is hybridising with the oligonucleotides-1 and -2 thus forming a three-stranded substrate complex, which launches the entropy-driven reaction based on a branch migration. The target NA links to the single-stranded domain on the substrate, which leads to a four-stranded intermediate (I1) creation and then to the I2 after rearranging. In the interim, the binding between oligonucleotide-2 and capture NA weakens and I2 dissociates and frees oligonucleotide-2. The released domain of the capture NA alleviates the bounding of Ru(bpy)_3_^2+^ modified oligonucleotide-3 DNA luminescence label forming the I3, which is rearranged and excludes oligonucleotide-1. At that time, some pairs of bases between target and capture NAs dissociate so that the final product remains and the target NA is regenerated to continue the reaction with the substrate, thus performing the ECL signal amplification on the electrode. The evaluation of the performance of the ECL biosensor was performed by the EIS and ECL methods [71]. The DNA tetrahedron-based ECL biosensor revealed a high specificity and sensitivity with a LOD of 2.67 fM. The enzyme-free entropy-driven basis of the ECL biosensor enables avoiding the employment of expensive reagents and performing large-scale screening. In addition, RdRp has been detected in human serum samples [71]. 

#### 2.1.3. Plasmonic-Based Biosensors 

Surface plasmon scattering over the interface of a thin metal layer (typically noble metals) and the dielectric is the basic principle of plasmonic biosensors [75]. In this approach, the refractive index changes of the environment adjacent to the metal layer of the sensor surface are monitored in real-time during target biocompound and the immobilised biosensing element interactions [76,77,78,79]. Surface plasmon resonance (SPR) is the foundation of most plasmonic biosensors [75,80]. Interactions take place in two ways on a surface appropriate for observing SPR-based signals: (i) bulk SPR signal and (ii) localised SPR (LSPR) signal. The distinction between SPR and LSPR is defined by the dimensions of employed plasmonic nanoparticles [81]. The effects are based on the surrounding media’s refractive index changes causing the SPR angle or spectral shifts. 

A plasmonic biosensor with two functions: the plasmonic photothermal (PPT) effect and LSPR sensing transmission, has been described to enable the creation of an alternative technique for SARS-CoV-2 virus recognition in which detection is given by the complementing hybridisation of NAs, one of which is immobilised on the gold nanoislands (AuNIs). To intensify the signal, the LSPR and PPT effects were combined. For the RdRp gene, the LOD is 0.22 pM in respiratory samples. On-site PPT enhancement on AuNIs-based chips can be used to differentiate the RdRp gene of SARS-CoV and SARS-CoV-2 [82].

Plasmonic biosensing provides several technological advantages, including the ability to combine SPR with electrochemical, electroluminescence, and fluorescence approaches [83]. Furthermore, nanomaterials were used to build the optical aperture and achieve extremely sensitive viral identification using the SPR method in combination with colorimetric and fluorescence-based techniques [75]. Plasmonic nanomaterials can be represented by metallic nanoparticles or graphene nanostructures [84,85,86,87].

### 2.2. Serological Tests

#### 2.2.1. Antibodies against SARS-CoV-2 tests 

The lateral flow immunoassay (LFIA) is a membrane strip with a sample well, a conjugate pad, test lines, and a control line. The conjugate pad includes the gold conjugates of SARS-CoV-2 antigen and rabbit antibody, the test lines are covered by anti-human IgG and IgM, respectively, whereas the control line is coated with anti-rabbit IgG. When the sample is added, IgG and IgM move toward the lines running through the conjugation pad, wherein the immunoglobulins interact with SARS-CoV-2 gold-covered antigens. The generated immune complexes attach to the test lines and couple with immobilised anti-human IgG and IgM, where rabbit gold conjugated antibodies adhere to the control line and interact with immobilised anti-rabbit IgG antibodies. Due to the simultaneous determination of IgG and IgM, the described serological approach has the major benefit of being able to diagnose COVID-19 at various phases of infection. IgG-IgM LFIA was revealed to be a reliable assay characterised by a sensitivity of 88.66%, specificity of 90.63%, and a testing time less than 15 min [88]. 

In the case of the enzyme-linked immunosorbent assay (ELISA), the SARS-CoV-2 antigen is deposited on the internal surface of multi-well polystyrene plates [89]. The patient sample is then added and left to incubate for an hour. Moreover, secondary antibodies coupled with an enzyme-reporter are appended. The secondary antibodies recognize and interact with specific antibodies present in the immune complex with the SARS-CoV-2 antigen [90]. The specific antibodies located on the surface are identified from colour changes (3,3′,5,5′-tetramethylbenzidine is used as chromogen) after adding the fully prepared substrate to the enzyme attached to the secondary antibody [91,92]. ELISA for the IgM detection has a sensitivity of 44.4% and specificity of 100%, whereas IgG has those 82.54% and 100% correspondingly. The combined IgM and IgG detection is characterised by the sensitivity of 87.3% [93]. Nevertheless, the method needs a test time of 2–5 h [94]. 

Chemiluminescence immunoassay (CLIA) is a label-based approach that involves the introduction of chemiluminescent labels or enzymatic tags, followed by the addition of a substrate based on luminol, which causes a chemiluminescence signal, which can be measured using a luminescence detecting device [95]. In one example of CLIA performed for COVID-19 diagnosis, as the capture agents, the conjugate of magnetic beads and recombinant N-proteins, is employed, alkaline phosphatase-labelled anti-immunoglobulin antibodies are utilised as the recognition probes, and as the chemiluminescent label, Lumigen alkaline phosphatase substrate 5, is utilised. This approach provides the sensitivity of 82.28% for both antibody types, as well as 97.5% for IgG and 81.25% for IgM [96]. Notably, the conjugate of magnetic beads and recombinant S-protein or SARS-CoV-2-specific open reading frame 1a/b proteins might be employed as capture agents [97]. 

##### Electrochemical Biosensors for Antibodies against SARS-CoV-2 Detection

An electrochemical paper-based analytical device for diagnosing COVID-19 (ePAD COVID-19) was reported for the detection of IgG and IgM [98]. As a substrate material, paper is used, which possesses such advantages as natural abundance, low cost [99], and it might be disposed of by cremation making it more proper for infectious disease testing. The ePAD consists of three parts, i.e., working, counter, and closing ePADs. To capture antibodies against SARS-CoV-2, S-protein containing RBD is immobilised on the working ePAD. The counter ePAD is manually wrapped to the working ePAD and topped with the closing ePAD, which is covered by a ferrocene redox probe for electrochemical detection. The registration of electrochemical response, i.e., signal decreasing after antigen-antibody complex formation, is performed by SWV. The approach was shown as rapid (30 min), with LOD of 1 ng/mL, the sensitivity of 100%, and the specificity of 90% for the method of the antibodies against SARS-CoV-2 detection in sera of patients. Moreover, the device can be used for the direct recognition of the S-protein [98]. 

A rapid (within seconds) advanced nanomaterial-based platform detecting antibodies against SARS-CoV-2 was described [100]. The platform represents electrodes made by 3D nanoprinting. Complex geometries, material combinations, and bespoke microstructures are all possible by 3D nanoprinting technology. This technique possesses such benefits as uncomplicated two-step manufacture, customisability, prototyping capability, and process control by computer-aided design software. The aerosol jet (AJ) fabrication technique employs an aerosol droplet flow for applying a range of nanomaterials with a resolution of 10 μm and it has been utilised to produce a variety of electronic devices [101]. The resulting 3D electrode is covered by nanoflakes of reduced-graphene-oxide covered by immobilised SARS-CoV-2 antigens (S1-protein and RBD). Further, the electrode is combined with a microfluidic tool and employed in a standard electrochemical cell. Antigen-antibodies interactions alter the impedance of the electrical circuit which is registered by EIS. The smartphone-based readout shows the LOD of antibodies against S1-protein and RBD are 2.8 fM and 16.9 fM, respectively. In addition, the immunosensor can be quickly (within a minute) regenerated by applying a low-pH for the antigen-antibody complex dissociation [100].

##### Ellipsometry- and SPR-Based Biosensors

Optical ellipsometry-based approaches provide a lot of possibilities for designing different affinity immunosensors [102]. In comparison to other SARS-CoV-2 detection methods (western blot, RT-PCR, ELISA, and indirect fluorescence), imaging ellipsometry has confirmed itself as a direct, non-destructive, rapid, label-free, and low-cost assay [103]. The kinetics of interactions between N-protein of SARS-CoV-2 immobilised on a self-assembled monolayer (SAM)-modified gold disks and antibodies against it were recently monitored using spectroscopic ellipsometry (SE) in its total internal reflection mode (TIRE) [104]. TIRE used phase shift measurement to detect biomolecule mass alterations at the solid-liquid interface. SE TIRE’s high sensitivity was achieved with the help of SPR, which allowed the simultaneous registration of two kinetic curves Ψ(*t*) and Δ(*t*) [105,106]. According to the mathematical model construction, it was found that the antigen-antibody complex is firmly bonded, and the complex formation has very tight orientation criteria [104].

##### Photoluminescence-Based Biosensors

**Figure 4:**
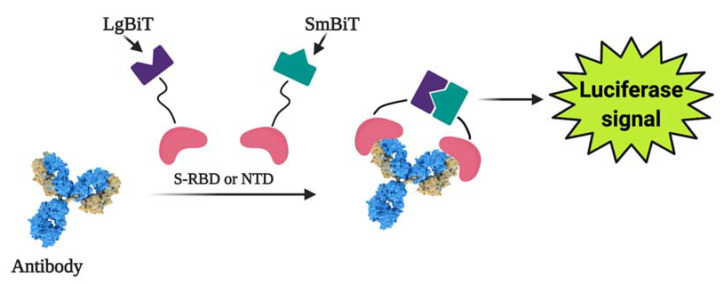
The general working concept of a split luciferase-based biosensor. Figure from [22].

Photoluminescence is a highly sensitive technology that can be used to create a variety of biosensors for detecting pathological cells [107] and virus-induced infections [108,109,110]. A split luciferase (spLUC)-based assay was designed that is proving to be a simple, quick (~5 min), accurate (~98%), low-volume specimen (1 µL per reaction), cheap, and quantitative technique to detect antibodies against SARS-CoV-2 S- and N-proteins [111]. Small BiT (SmBiT) and large BiT (LgBiT) nanoluciferase (NanoLuc) fragments [112] were fused to viral protein antigens to design the biosensor. As immunoglobulins have two antigen-linking sites, incubating a 1:1 mix of SmBiT and LgBiT with blood serum results in the pairing of LgBiT with one antigen-linking spot and SmBiT with another spot. When LgBiT and SmBiT fragments are fixed, the NanoLuc enzyme is reduced, allowing for subsequent luminescence-based identification [111] (Figure 4).

Considering SARS-CoV-2 carriers have antibodies directed predominantly towards S- and N-protein epitopes, sensors based on these viral proteins were developed [113,114]. The sensor based on the N-protein-based was created using NTD linking with NanoLuc fragments, whereas in the sensor based on the S-protein RBD was employed. The proportion of immunoglobulin concentration to signal intensity was described using ordinary differential equation modelling, and it was revealed that the concentration-signal ratio is linear. The sensor has an 89% and 98% sensitivity to S-protein to N-protein, respectively [111]. The spLUC technique has key characteristics bypassing the arduousness of multi-step ELISA-based analyses, which makes the spLUC suitable for point-of-care (POC) diagnostics [115]. The reagents employed in the spLUC test are highly resilient against lyophilisation for storage and transport, as well as able to quickly detect immunoglobulins directly from clinical specimens. Another advantage of the assay is its modularity, which allows adjusting to the immune response to practically any viral infection with distinguished antigens [111]. 

#### 2.2.2. SARS-CoV-2 Viral Particles and Structural Proteins Tests

The LFIA-based ‘COVID-19 Ag Respi-Strip (CORIS)’ test uses nitrocellulose-based membrane technology and colloidal AuNPs coupled with monoclonal antibodies against the SARS-CoV-2 N-protein. With a sensitivity of 30.2% and a specificity of 100%, the approach can determine the antigen in a sample within 15 min [116]. 

Fluorescent dyes (fluorophores) are employed in fluorescent immunoassays (FIA) to evaluate the target signal through fluorescence microscopy. The ‘standard F COVID-19 Ag FIA’ test for recognition of SARS-CoV-2 N-proteins is an instance of an FIA-based technique. It includes a cassette on which a pre-extracted sample combines with a monoclonal antibody against SARS-CoV-2, and the fluorescence analyser measures the amount of fluorescence caused by the formation of antibody-antigen complex after incubation. The test takes 30 min and has a sensitivity of ~47% [117]. Fluorescence immunochromatography (FIC) [118], CLIA [119], and lateral flow assay (LFA) [120] are also used to determine N-protein.

##### Electrochemical Biosensors for Viral Particles and SARS-CoV-2 Structural Proteins Detection

Reagent-free detection of VPs employing a chip sensor-modified electrode was reported [121]. This method is based on the electrode-tethered sensor which carries an analyte-binding antibody imaged on a negatively charged DNA linker equipped with an associated redox probe. After the application of positive potential, the chip moves towards the surface of the electrode. The VPs and structural proteins can be detected by chronoamperometry as these particles increase the flow resistance of the sensor. The sensor includes an analyte-recognising antibody connected to a firm, negatively charged DNA-based linker [122,123]. To monitor the interaction of the chip with the electrode surface, a ferrocene redox probe is connected to the DNA linker [124]. Further electron transfer and ferrocene oxidation occur with a characteristic time constant, *t*. It was shown that this method is effective for SARS-CoV-2 and S-protein detection in test samples and patient saliva within 5 min [121].

Virus-imprinted chips (VIC) is an impedimetric sensing platform, which was built employing carbon nanotubes (CNT)/WO_3_-screen printed electrodes for imprinting the SARS-CoV-2 VPs into the polymeric matrix, thereby producing binding sites [125]. EIS measurements were performed in a double-mediating system (mixture of potassium ferrocyanide(III) (FCN) and 2,6-dichlorophenolindophenol (DCIP)). The sensor revealed high selectivity over Influenza A and Influenza B viruses, human coronaviruses (hCoVs)-OC43, NL63, and 229E, and the MERS-CoV. Moreover, LOD and limit of quantification were 57 and 175 pg/mL, respectively. The sensor was used in clinical samples acquired from nasopharyngeal swabs from SARS-CoV-2 suspected patients, thereby making it suitable as a point-of-care (POC) device [125]. 

A nasopharyngeal swab is the main method for sample collection. The conventional RT-PCR for SARS-CoV-2 detection requires additional steps of samples transfer into solution and RNA extraction. In contrast, the reported cotton-tipped electrochemical immunosensor [126] includes the integration of the sample collection and detection. The combination is achieved by using cotton padding to coat screen-printed electrodes. N-protein is immobilised on the carbon nanofiber modified screen-printed electrode. The antigen further is functionalised by diazonium electrografting and activated by N-hydroxysuccinimide and 1-ethyl-3-(3-dimethylaminopropyl)-carbodiimide hydrochloride. Viral antigen detection is performed by a competitive method using a fixed concentration of antibodies against N-protein in solution. For signal registration, the SWV technique is applied. The LOD of the developed immunosensor is 0.8 pg/mL. The immunosensor showed no cross-reactivity with antigens from other viruses including influenza A and human coronavirus, demonstrating good selectivity. Signal registration is performed by a potentiostat and tracked by a smartphone [126]. 

Recently, a voltammetry-based sensor for SARS-CoV-2 S-protein detection was reported [127]. The sensing system included bovine serum albumin, antibody against SARS-CoV-2 S-protein and functionalised graphene oxide modified glassy carbon electrode (fGO/GCE) or screen-printed electrode (fGO/SPE). The antibody-antigen interaction was analysed by SWV in a presence of a redox probe. The method was characterised with 92.5% specificity and 93.3% sensitivity with a testing time of 5–35 min depending on sample type. GCE- and SPE-based sensors could define 1 ag/mL of S-protein in saliva or oropharyngeal swab and showed a dynamic response to S-protein in a 1 ag/mL–10 fg/mL range. Thus, the developed immunosensor has a potential for the COVID-19 diagnosis in real samples [127].

Since the protein is detected on the signal transducing surface surface in biosensors, the development of such sensors necessitates the design of a surface with sufficient protein recognition capabilities. Molecularly imprinted polymers (MIPs) can be very suitable for this purpose [59,128,129,130,131]. Molecularly imprinted sensors have the benefit of low cost and high stability, and they are usually made from protein-imprinted polymers like polypyrrole [132,133] and some other electrochemically deposited polymers [134,135,136]. Different signal determination methods can be utilised in the development of MIP-based sensors, but the most common ones are potentiodynamic electrochemical techniques [132] and quartz crystal microbalance (QCM) based approaches [137,138]. MIPs can be designed for low molecular weight compounds, making their development and implementation in sensor design reasonable [54,139]. The technique was also shown to be effective in determining the presence of some viral proteins [132]. It should be emphasised that MIP-based sensors can detect even certain DNA-based oligomers [129], making MIP-based sensors appealing for DNA- and potentially for RNA-fragment detection. 

Moreover, owing to the relatively low price compared to that of antibodies, MIPs might replace antibodies in the design of bioanalytical systems including immunosensors. The very first application of molecular imprinting technology for the SARS-CoV-2 was done by Parisi et al. [140]. The mentioned study described the development of so-called ‘monoclonal-type plastic antibodies’ based on MIPs for selective binding of the S-protein. One of the most important advantages of electrochemical methods is the ability to miniaturize sensors for portable analytical devices and minipotentiostats compatible with smartphones [141,142]. Exactly like those electrochemical methods for portable analytical devices and minipotentiostats compatible with smartphones as developed by V. Syritski research group [143,144]. Raziq et al. [143] developed the MIPs-based technology used to produce an electrochemical sensor for detecting the SARS-CoV-2 N-protein. The sensor was a disposable slim film electrode selective for the SARS-CoV-2 N-protein. DPV detected the line electrochemical signal from N-protein up to 111 fM, with a detection limit of 15 fM [143]. The SARS-CoV-2 N-protein (ncovNP) sensitive MIP sensor was designed by electrochemical deposition of poly-m-phenylenediamine on the gold-based thin-film metal electrode (Au-TFME) [143]. The optimisation steps of the sensor were characterised by CV. Meanwhile, the rebinding of SARS-CoV-2 N-protein on the sensors was studied by DPV in the solution of 1 M KCl containing a redox probe K_3_[Fe(CN)_6_]/K_4_[Fe(CN)_6_]. The obtained results demonstrated the linear increase of the sensor response with increasing ncovNP concentration. The feasibility of sensor performance in clinical samples was tested. For this purpose, they analysed the samples prepared from nasopharyngeal swab specimens. In the following study, the same research group described an electrochemical sensor that was capable of giving a satisfactory performance with a reaction time of 15 min [144]. However, in this study, MIP was formed by electrochemical deposition of poly(3-aminophenylboronic acid) on the same disposable Au-TFME chip modified with the aminothiophenol. The LOD of SWV method was only 15 fM of SARS-CoV-2 S-protein subunit S1 in the phosphate-buffered saline sample. Genetically engineered SARS-CoV-2 RBD protein was imprinted in *o*-phenylenediamine and deposited on a macroporous gold screen-printed electrode [145]. The LOD of the suggested EIS method was 0.7 pg/mL. The polypyrrole in presence of SARS-CoV-2 S-protein electrochemically was deposited on the platinum electrode [146]. The suggested method is unique and different from the abovementioned studies because no redox probe was used during evaluation by pulsed amperometric detection. Hence, the evaluation method was based on the conductivity changes of the polypyrrole layer in presence of different concentrations of SARS-CoV-2 S-protein. The MIP sensor based on chemically polymerized 3-aminophenyl boronic acid and pyrrole decorated with graphene oxide with SARS-CoV-2 antigen imprints was also developed [147]. The MIP was deposited on a glassy carbon electrode and used CV, DPV, amperometry, and EIS methods. The LOD of the proposed voltammetric and amperometric methods was 0.326 fg/mL and 11.32 fg/mL.

The MIPs were applied for the determination of SARS-CoV-2 proteins and by methods other than electrochemical. The SPR method employing the highly affine MIPs for the recognition of the RBD of SARS-CoV-2 S-protein was developed by Bognar et al. [148]. The SPR sensor modified with MIP imprinted peptide epitope was obtained by electrochemical deposition of polyscopoletin film. The efficiency of three peptide epitopes was compared. The obtained results proved the efficiency of epitope-imprinted polyscopoletin ligands that bound the SARS-CoV-2 S-protein RBD with higher affinity than its natural target ACE2. Another important note is that the obtained sensor was selective over the influenza A virus. A SARS-CoV-2 S-protein (S1 subunit, His-Tag) was imprinted in chemically polymerized acrylamide-based MIP [149]. The designed acrylamide-based MIP was coated on the plastic optical fiber-based SPR sensor. Obtained results proved that the prototype sensor was capable of detecting the S-protein of SARS-CoV-2 in various solutions and the virions as well. 

Nanostructuring the electrode improves the functionality of the electrochemical biosensor by increasing the rate of the electrochemical reaction, owing to an increase in the electrode surface area to analyte volume ratio. For instance, it was reported that cobalt-functionalised TiO_2_ nanotubes (Co-TNTs) with a high surface-to-volume ratio detect biomarkers related to tuberculosis [150,151]. Owing to the Co ions reduction and biomarker oxidation, a Co-biomarker complex is formed at a predetermined bias voltage. This phenomenon underlies the proposed sensing mechanism [152]. Co-TNT by way of a sensing material for the electrochemical-based detection of SARS-CoV-2 RBD of S-protein (S-RBD) was reported [152]. TNTs were made using a one-step electrochemical anodising technique that was simple and cost-effective. Whereas the incipient wetting approach (wet ion-exchange process) was used for Co-functionalisation. Custom-built Co-TNT packaged printed board setup with a plugged potentiostat was used for the electrochemical detection of S-RBD protein. The circuit board includes a copper clamp holding the Co-TNT, which was grown over the Ti-based sheet. The Co-TNT upward side serves as a working electrode. As the bottom side of Co-TNT acts as a counter electrode [153]. Amperometry was used to determine the concentration of the analyte between the electrodes [152]. It was found that the sensor can detect the S-RBD protein rapidly (~30 s) in the range of concentrations from 14 to 1400 nM, which can be explored for developing a POC detection of SARS-CoV-2 in nasal secretions and saliva samples. With LOD down to 0.7 nM, the ratio between sensor response and the protein concentration was revealed as linear [152].

Recently reported S1-protein detection method uses mammal Vero cells with the human chimeric S1-antibody electro-inserted into them. The method is also known as ‘Molecular identification through membrane engineering’. This technique is a cell-based approach for determining analytes based on the specific interaction of target compounds with cellular biorecognition elements whose surfaces are modified by electrochemical insertion of target-specific antibodies [154]. The coupling of target compounds to the electrochemically entrapped antibodies [155] caused a distinct and observable alteration in the electric characteristics of the biorecognition components, particularly the hyperpolarisation of the engineered cell membrane [156,157]. It was shown that the linking of the SARS-CoV-2 S1-protein to the corresponding antibody leads to a significant and selective change in the bioelectric characteristics of the membrane-engineered cell [154]. The actual availability (binding and/or uptake) of analytes is determined by changes in the membrane potential and other electric properties of the cells. The measurements, based on the principle of the bioelectric recognition assay (BERA) [158,159], were performed by a customised multichannel potentiometer. The assay was also integrated with a customised handheld read-out device that could be controlled using a smartphone or tablet [154]. The biosensor is characterised by a rapid output of the result (~3 min) with a LOD of 1 fg/mL and a semi-linear response range between 10 fg and 1 g/mL. Moreover, cross-reactivity vs. SARS-CoV-2 N-protein was not found. Furthermore, the sensor’s sensitivity suggests that it might be used for SARS-CoV-2 detection in patient saliva samples [154].

##### Nanoplasmonic-Based Biosensors

Diagnosis early in infection is limited to the detection of viral nucleic acid or antigen in nasopharyngeal swabs or saliva samples. Recently, a fast direct nanoplasmonic surface resonance (nPSR) based SARS-CoV-2 VPs detection and quantitation approach was developed [160]. The previously reported plasmonic nanocup array sensor chip manufacture method [161,162] allows for massive production with high homogeneity and repeatability reproducibility. Transmission light spectroscopy or imaging can easily monitor the shift of the plasmon resonance wavelength and intensity on the virus capture sensor surface due to the specifically built periodic nanostructures [163,164]. Thus, the nPSR chips can be combined with a micro-well plate, and measurements can be performed in both conventional microplate readers and a POC tool [161,162]. The chip-in-micro-well sensor was designed to detect varied concentrations of whole VPs in a direct multichannel manner [160]. Antibodies against SARS-CoV-2 were immobilised on the surface of the nPSR array sensor chip to capture the virus by binding to S-proteins on its surface. The primary absorption and differential spectra of consecutive phases of sensor modification showed noticeable alterations. A specific resonance wavelength of 640 nm can be found in the absorption spectra of the nanocup array chip. The nPSR sensor can detect up to 370 VP/mL SARS-CoV-2 pseudovirus with suitable antibody functionalisation on its surface. This assay might be used to determine SARS-CoV-2 viral concentrations in the range of 102–107 VP/mL with the simultaneous detecting of diluted reference samples on the same microplate reader [160].

The real-time monitoring of the dynamic binding curves of SARS-CoV-2 on the nPSR sensor handle and novel device controlled by smartphone app was reported [160]. The nPSR sensor chip was placed into a cartridge developed for the portable tool. Then the chip was functionalised, and the VPs detection was performed in the same way as for the microplate reader. After placing the functionalised chip cartridge into the gadget, a smartphone application logged the dynamic curves in real-time. The POC tool can rapidly (within 15 min) directly recognise the SARS-CoV-2 in a sample in a concentration range of over 0 up to 6.0 × 10^6^ VP/mL. Furthermore, the POC quantification limit is around 4000 SARS-CoV-2 VPs and can be enhanced to be comparable with the microplate reader method.

##### Field-Effect Transistor Based Biosensors

**Figure 5:**
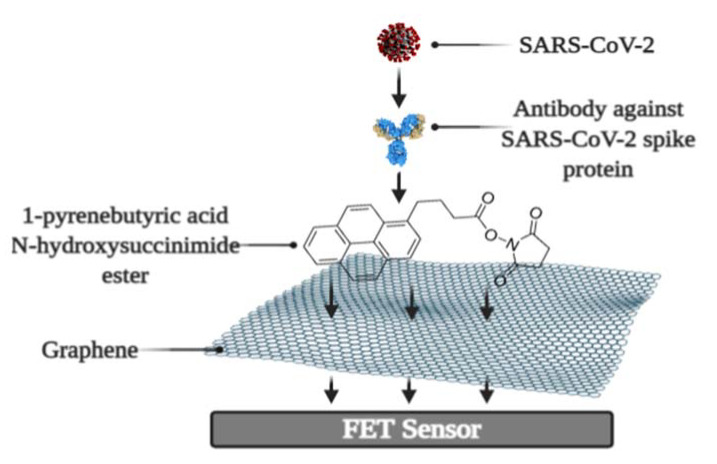
Schematic representation of FET immunosensor. Figure from [22].

It was recently revealed that a biosensor based on field-effect transistors (FET) detects SARS-CoV-2 in patient samples in real-time [165]. The FET graphene plates were coated with an antibody against the SARS-CoV-2 S-protein. The antibody was attached to the biosensor surface using the N-hydroxysuccinimide ester of 1-pyrenebutyric acid (Figure 5). The effectiveness of the immunosensor was tested using a cultivated virus, viral antigen, and nasopharyngeal swab samples from a virus carrier. The LOD was 1.6 × 10^1^ pfu/mL and 2.42 × 10^2^ copies/mL in culture medium and clinical specimens, respectively [165].

##### Quartz Crystal Microbalance Based Biosensors

Biosensors may be effectively developed using QCM [138]. In the QCM-based technique, the viral S-protein binds to the designed quartz crystal surface functionalised with SAM. The determination of proteins is based on utilising surface features such as hydrophobicity, which is one of the fundamental qualities of the analytical system’s active surface since an increase in surface wettability leads to a higher surface concentration of proteins [166,167]. SAMs with a wide range of hydrophobicity, regulated by functional groups on a surface, were researched and designed for this purpose [168]. SAMs with terminal carboxyl and methyl groups have been proven to be the most reliable for highly specific linking with the SARS-CoV-2 S-protein [169]. The fundamental operating concept of the QCM is to increase the adsorbed mass while decreasing the frequency of the quartz crystal vibrations [170]. As a result, QCM-based procedures provide for quick, sensitive, and label-free testing [170,171]. The proposed technique may be used for label-free, real-time detection with a sensitivity of up to ng in oral swab samples [169].

### 2.3. Other Biosensor Based Tests

#### 2.3.1. Determination of Reactive Oxygen Species

CoVs have been shown to increase viral replication in lung host cells by inducing reactive oxygen species (ROS) within mitochondria [172]. In SARS-CoV^3L^ proexpressing cells, ROS concentrations were significantly increased [173]. It is associated with high ROS levels with an aim of the activation of SARS-CoV 3a-induced NLRP3 inflammasome [174]. This is due to the viral infection which activates the generation of ROS [175].

The COVID-19-stimulated ROS detector is an electrochemical ROS/H_2_O_2_ device [176]. This gadget incorporates a sensor built from a multi-wall carbon nanotube (MWCNT) on the tip of steel needles, as well as an incorporated movable automated real-time electrochemical based monitor. The key functioning principles are the dipping of the electrode in the sputum and the registering signals of ROS. CV was used to determine ROS level intensity. The electrochemical ROS detection test is quick (less than 30 s), with lower than 500 μL sample volumes with an accuracy of over 97%. Moreover, the assay can be executed in vivo, skipping the specimen preparation step [176].

#### 2.3.2. Nanomaterial Based Sensor for the Diagnosis of COVID-19 in Exhaled Breath

Nanomaterial-based sensor arrays with multiplexed functionalities were designed for the diagnosis of COVID-19 from exhaled breath. The sensors are made up of several AuNPs coupled to organic ligands, resulting in a diversified sensitive layer. The layer can expand or shrink in response to volatile organic compounds (VOCs), which leads to electrical resistance alterations [177]. Thus, the organic compounds bind VOCs, while electrical conductivity is provided by inorganic nanoparticles [178,179]. Released VOCs get into the sensing surface and interact with the functional groups covering the inorganic elements, resulting in volume alteration (expansion/shrinkage) in the nanomaterial layer followed by conductivity changes [178]. The conductivity variations occur even when no steric alterations take place on the sensing layer, owing to VOC-induced charge transfer toward/from the inorganic nanomaterial [178,180]. The array consists of eight AuNPs-based sensors, and it is integrated with electronic circuitry and equipped by an exhaled breath collecting sample device. A mix of COVID-19-related VOCs interacts with the sensors when the breath goes through the array resulting in a series of electrical resistance signals plotted vs. time. COVID-19 markers are obtained using software-based machine learning approaches that examine the pattern of signal response [177].

Owing to the chemical variety of the functional groups capping the AuNPs, different sensors can be employed as a range of cross-reactive semi-selective sensory elements that mimic the sensing processing mechanism of natural human olfactory systems [178,181,182]. The versatility, as well as the ability to employ pattern identification and machine learning algorithms to train it to recognise a wide range of chemical patterns in various settings and for diverse purposes, is the main benefit of the nanomaterial-based sensor array [178,181].

## 3. Conclusions

It is critical to be able to diagnose COVID-19 quickly to prevent the spread of the infection. There are several limits of conventional molecular and serological approaches. A considerable sample processing time is required for molecular techniques, which also necessitates the use of specialised and costly facilities. While serological tests avoid these drawbacks, they are less sensitive and restricted in their ability to diagnose COVID-19. Biosensors provide the promise of replacing current bulky and complicated processes with a contemporary, easy, portable, accurate, and sensitive alternative.

In this paper, we overviewed various biosensors for COVID-19 diagnosis which were classified into molecular and serological types (Table 2). The precision of molecular biosensors based on the registration of NA hybridisation was demonstrated, however, they still have limitations due to the need for an amplification step. The antisense oligonucleotides electrochemical biosensor is promising, owing to its low LOD (6.9 copies/μL), short testing time (~5 min), and avoidance of using labels. Antigen-antibody affinity is the most common analytical signal source in serological sensors. The highest sensitivity (100%) belongs to ePAD for COVID-19 diagnosis, the benefit of which is that it is also a naturally abundant, cheap, and disposable paper-based substrate material. The AJ electrochemical biosensor was shown as the most rapid, enabling detection of antibodies against SARS-CoV-2 S1-protein or RBD within only seconds. MIP-based electrochemical sensors have one of the lowest LOD (up to fM) among the discussed sensors. In addition, MIP-based sensors are characterised by higher stability than protein-based sensors. Another attractive label-free diagnostic tool is the ROS detection approach, which has a high sensitivity (>97%) despite a relatively small sample volume. Irrespective of the undetermined sensitivity, the SE/SPR-based approach allowed key inferences to be drawn regarding the antigen-antibody complex structure and the kinetics of its formation, which is useful for the development of novel immunosensors. 

Based on the considered research, we can conclude that electrochemical and optical signal registration are the most often utilised signal registration methods. The reviewed biosensors demonstrate a strong trend to build analytical systems that are easy to use, owing to the exclusion of extra steps in sample preparation and the employment of additional labelling compounds. Electrochemical sensors detecting the interaction of target bio compounds with complementary recognising chemicals adsorbed on the working surface are ideally suited to these conditions. Furthermore, because of their low cost, simplicity, and mass manufacturing capacity, electrochemical biosensors have the most widespread application for biomedical purposes.

While great effort has been done to explore the features of the SARS-CoV-2 and the techniques used to detect it, there is still a need for further development and enhancement of diagnostic approaches that avoid the flaws of existing methods and take advantage of new ones.

## Figures and Tables

**Table 2:** Summary table of the reviewed biosensors.

Biosensor			Biorecognition Element	Signal Source	Registration Methods	Label Need	LOD	Sensitivity	Test Time	Sample	Reference
Molecular tests											
Electrochemical		Calixarene functionalised graphene-based	Capture NA	RNA hybrid.	DPV-signal	Label NA	200 copies/mL	85.5%	-	Clinical	[62]
		RCA	Circular DNA template	Amplicons hybrid.		Redox dye	1 copy/μL	-	~2 h	Nasopharyngeal swabs	[68]
		Antisense oligonucleotides	ssDNA specific for N-gene	ssDNA-RNA hybrid.	Current-voltage signal conditioning circuit	Label-free	6.9 copies/μL	231 copies/μL	~5 min	Nasal swab/saliva sample	[69]
ECL			Capture NA specific for RdRp	RNA hybrid.	EIS and ECL	Luminescence label	2.67 fM	-	-	Serum	[71]
Plasmonic			Capture NA		PPT+LSPR	Label-free	0.22 pM	-	-	Respiratory samples	[82]
Serological tests											
Antibodies against SARS-CoV-2 tests	Electrochemical	ePAD	S-protein containing RBD	Antibody-antigen affinity	SWV	Redox probe	1 ng/mL	100%,	~30 min	Serum	[98]
		AJ-based	S1-protein and RBD		EIS		2.8 fM (Ab against S1); 16.9 fM (Ab against RBD)	-	Within seconds		[100]
	Spectroscopic ellipsometry		N-protein		TIRE+SPR signals	Label-free	-	-	-		[104]
	Optical		S- or N-protein		Photoluminescence	SmBiT and LgBiT	-	89% (S-sensor); 98% (N-sensor)	-		[111]
Structural SARS-CoV-2 proteins or whole virus tests	Electrochemical	Electrode-tethered	Antibody against S-protein		Chronoamperometry	Redox probe	-	-	~5 min	Saliva	[121]
		VIC	CNT/WO_3_ modified electrode selective to VPs	VP binding	EIS	FCN/DCIP	57 pg/mL	-	-	Nasopharyngeal swabs	[125]
		Cotton-tipped	Antibody against N-protein	Antibody-antigen affinity	SWV	Redox probe	0.8 pg/mL	-	-		[126]
		fGO/GCE fGO/SPE	Antibody against *S*-protein			Redox probe	1 ag/mL	93.3%	5–35 min	Saliva/oropharyngeal swab	[127]
		MIP-based	Selective to N-protein	Antigens- binding	DPV	Redox probe	15 fM	-	-	Nasopharyngeal swabs	[143]
			Selective to S1-subunit		SWV		15 fM	-	15 min	-	[144]
			Selective to S-RBD		EIS		0.7 pg/mL	-	-	-	[145]
			Selective to S-protein		CV, DPV, amperometry, EIS		Volt: 0.326 fg/mL Amp: 11.32 fg/mL	-	-	-	[147]
			Selective to S-RBD		SPR		-	-	-	-	[148]
		TiO_2_ nanotube-based	Co-TNTs	S-RBD oxidation	Amperometry	Label-free	0.7 nM	-	~30 s	Nasal secretions and saliva samples	[152]
		Cell-based	Antibody against S1-protein	Antibody-antigen affinity	BERA		1 fg/mL	-	~3 min	Saliva	[154]
	nPSR		Antibody against S-protein		SPR		370 vp/mL 4000 vp/mL (POC)	-	~15 min (POC)	Nasopharyngeal swabs/Saliva	[160].
	FET		Surface properties alterations		FET current response		242 copies/mL	-	-	Nasopharyngeal swab	[165]
	QCM			S-protein binding	Change of QCM resonance frequency		-	-	-	Oral swab samples	[169]
Other tests											
ROS detection			MWCNT electrode	ROS level	CV	Label-free	Sputum sample vol. < 500 μL	>97%	~30 s	Sputum	[176]
Multiplexed nanomaterial-based			Organic ligands	COVID-19 related VOCs	Electrical/electrochemical		-	-	-	Exhaled breath	[177]

## Data Availability

Not applicable.
